# Preoperative Fibrinogen-to-Albumin Ratio as Potential Predictor of Bladder Cancer: A Monocentric Retrospective Study

**DOI:** 10.3390/medicina58101490

**Published:** 2022-10-19

**Authors:** Biagio Barone, Luigi Napolitano, Pasquale Reccia, Luigi De Luca, Simone Morra, Carmine Turco, Alberto Melchionna, Vincenzo Francesco Caputo, Luigi Cirillo, Giovanni Maria Fusco, Francesco Mastrangelo, Francesco Paolo Calace, Ugo Amicuzi, Vincenzo Morgera, Lorenzo Romano, Massimiliano Trivellato, Gennaro Mattiello, Enrico Sicignano, Francesco Passaro, Gianpiero Ferretti, Gaetano Giampaglia, Federico Capone, Celeste Manfredi, Felice Crocetto

**Affiliations:** 1 Department of Neuroscience and Reproductive and Odontostomatological Sciences of University of Naples “Federico II”, 80131 Naples, Italy; 2 Unit of Urology, Department of Woman, Child and General and Specialized Surgery, University of Campania “Luigi Vanvitelli”, 80131 Naples, Italy

**Keywords:** fibrinogen-to-albumin ratio, bladder cancer, biomarker

## Abstract

*Background and objective:* Fibrinogen and albumin are two proteins widely used, singularly and in combination, in cancer patients as biomarkers of nutritional status, inflammation and disease prognosis. The aim of our study was to investigate the preoperative fibrinogen-to-albumin ratio (FAR) as a preoperative predictor of malignancy as well as advanced grade in patients with bladder cancer. *Materials and Methods:* A retrospective analysis of patients who underwent TURBT at our institution between 2017 and 2021 was conducted. FAR was obtained from preoperative venous blood samples performed within 30 days from scheduled surgery and was analyzed in relation to histopathological reports, as was the presence of malignancy. Statistical analysis was performed using a Kruskal–Wallis Test, and univariate and multivariate logistic regression analysis, assuming *p* < 0.05 to be statistically significant. *Results:* A total of 510 patients were included in the study (81% male, 19% female), with a mean age of 71.66 ± 11.64 years. The mean FAR was significantly higher in patients with low-grade and high-grade bladder cancer, with values of 80.71 ± 23.15 and 84.93 ± 29.96, respectively, compared to patients without cancer (75.50 ± 24.81) (*p* = 0.006). Univariate regression analysis reported FAR to be irrelevant when considered as a continuous variable (OR = 1.013, 95% CI = 1.004–1.022; *p* = 0.004), while when considered as a categorical variable, utilizing a cut-off set at 76, OR was 2.062 (95% CI = 1.378–3.084; *p* < 0.0001). Nevertheless, the data were not confirmed in the multivariate analysis. *Conclusions:* Elevated preoperative FAR is a potential predictor of malignancy as well as advanced grade in patients with bladder cancer. Further data are required to suggest a promising role of the fibrinogen-to-albumin ratio as a diagnostic biomarker for bladder tumors.

## 1. Introduction

Bladder cancer (BC) is one of the most diagnosed genitourinary cancers worldwide, accounting for 3% of global cancer diagnoses with over 550,000 cases diagnosed in 2018 [1]. In addition, BC ranks thirteenth in terms of deaths, with an overall mortality age-standardized ratio (ASR) of 3.2 per 100,000 in men and an ASR of 0.9 per 100,000 in women; this figure is even higher in developed countries (ASR = 4.5 per 100,000 in men and ASR = 1.1 per 100,000 in women) [2]. Despite the incidence of BC having decreased in recent years, it remains high in the elderly and in smokers [3,4,5]. BC can be divided, based on the degree of invasion, into non-muscle-invasive bladder cancer (NMIBC) and muscle-invasive bladder cancer (MIBC), with the first accounting for ~80% of total diagnosed BC [2,6]. A further division can be made, according to the World Health Organization, between low-grade (LG) and high-grade (HG) BC, which has implications for the stratification and management of patients affected [7,8].

According to the European Association of Urology (EAU) guidelines, for early-stage BC and NMIBC, transurethral resection of bladder tumor (TURBT) followed by intravesical chemotherapy or immunotherapy represents the treatment of choice; conversely, for a tumor invading the muscle layer, which determines the progression to MIBC, radical cystectomy (RC) and urinary diversion represent the gold standard [9,10]. Due to the morbidity and mortality related to this disease, with important repercussions especially in the progression from NMIBC to MIBC, it is of utmost importance that researchers identify a simple, economical, and promptly available clinical biomarker for the early identification and classification of BC patients [11].

Several studies suggest that hypercoagulability, inflammation, and malnutrition promote the occurrence, development, recurrence, and metastasis of different tumors and are associated with poor treatment outcomes [12,13]. Fibrinogen is a 340 KDa glycoprotein synthesized by hepatocytes and is involved into the coagulation process via conversion to fibrin, mediated by the activated thrombin. In addition to its role as a risk factor in various thrombotic diseases and as a marker of inflammatory state (due to its acute-phase protein nature), recent evidence has demonstrated that fibrinogen could accumulate at tumor sites and play an important role in the progression to malignancy [14,15,16,17]. Albumin, which is also produced by the liver and presents anti-inflammatory and antioxidant activity, is widely used as an indicator of nutritional status [18]. In addition, pretreatment serum albumin levels provide prognostic significance and risk stratification in cancer patients [19]. Based on these premises, the aim of our monocentric and retrospective study was to evaluate the role of a novel biomarker, the fibrinogen-to-albumin ratio (FAR), as a potential preoperative predictor of BC grade and stage, in addition to its proven efficacy as a marker related to the nutritional, coagulation, and inflammatory status of cancer patients [20,21,22].

## 2. Materials and Methods

We retrospectively analyzed clinical and laboratory data retrieved from patients who underwent TURBT at our University Hospital “Federico II” in Naples between January 2017 and January 2021. According to the European Association of Urology (EAU) guidelines, patients underwent TURBT after a cystoscopy that suggested a potential malignancy [9]. The research was conducted according to the Declaration of Helsinki on ethical principles for medical research involving human subjects. All patients provided written informed consent for the inclusion of their data in a database and for their use for scientific research purposes. No Ethics Committee approval was needed based on the study design. The inclusion criteria comprised: patients aged >18 years with a previous cystoscopy performed within two months suggesting a potential malignancy. The exclusion criteria were: previous BC diagnosis, active hematogenous or urinary infection, acute systemic inflammatory conditions (such as autoimmune diseases), coagulation-related diseases, hepatic dysfunction, and any other potential tumor at the time of cystoscopy. Routine venous blood samples were obtained within 30 days before scheduled surgery at our institutions, while demographic information was retrieved from medical records. FAR was calculated as the ratio between fibrinogen and albumin values. The stage and the grade of BC were determined by examining the tissue sample obtained via the TURBT procedure.

### Statistical Analysis

Descriptive statistics were reported as means and standard deviations for continuous variables, while frequencies and percentages were reported for categorical variables. The normality of the data was assessed using the Kolmogorov–Smirnov test before we proceeded to further analysis. A Kruskal-Wallis Test was utilized to evaluate variables in relation to the histopathological results (negative, LG, or HG), while univariate and multivariate logistic regression analyses were utilized to evaluate statistically significant variables in relation to the presence of any-grade BC. The receiver operating characteristic (ROC) curve and the Youden index were utilized to provide the optimal cut-off for FAR. Statistical analysis was conducted using IBM SPSS software (version 25, IBM Corp, Armonk, NY, USA), considering *p* < 0.05 to be statistically significant.

## 3. Results

A total of 510 patients were retrospectively involved in the study. The descriptive characteristics and preoperative laboratory data are reported in Table 1. The median age at the time of surgery was 73 years, while a preponderance of male patients compared to female patients was reported (81% versus 19%). A total of 15.3% of patients were diabetic, while 11.4% of patients reported some grade of chronic kidney disease (eGFR < 90 mL/min). In the histopathological analysis performed after TURBT, a total of 340 (66.7%) BCs were reported, accounting for 152 (29.8%) low-grade BCs and 188 (36.9%) high-grade BCs. Among negative results, which accounted for 33.3% of cases, chronic cystitis and focal atrophies were mostly founded. Finally, among the 340 BCs, 59 (17.8%) were muscle-invasive and required radical cystectomy. Differences in the preoperative data among different BC gradings are reported in Table 2. Age was increasingly higher from negative results to high-grade BC, with a median age of 70 years for negative results, 72 years for low-grade BC, and 75 years for high-grade BC (*p* < 0.0001). Mean preoperative hemoglobin, conversely, showed decreasing values from negative results to high-grade BC, with a median of 14.5 g/dL for negative results, 14 g/dL for low-grade BC, and, finally, 13.6 g/dL for high-grade BC (*p* < 0.0001). Although median creatinine differences were statistically significant among negative (0.9 mg/dL), low-grade (0.9 mg/dL) and high-grade (0.92 mg/dL) BC, we did not observe a clinically relevant value in those findings, considering the tight variation among the categories investigated (*p* = 0.008). Regarding albumin, patients with negative results reported a median value of 4.4 g/dL, low-grade BC patients reported 4.4 g/dL, and high-grade BC patients reported 4.3 mg/dL (*p* = 0.021). Median ALT was, similarly, 19 mg/dL in patients with negative results, while it was 17 mg/dL and 16 mg/dL in low-grade and high-grade BC patients, respectively (*p* = 0.004). No statistically significant difference was reported for fibrinogen values alone, or for the other variables retrieved. Conversely, FAR increased linearly from negative results to LG and HG BC, showing, respectively, median values of 70.55, 78.2, and 81.01 (*p* = 0.016). Among the categorical variables, the only statistically significant results were obtained for gender (which was preponderantly unbalanced towards male) (*p* = 0.022) and diabetes (*p* = 0.027).

**Figure 1:**
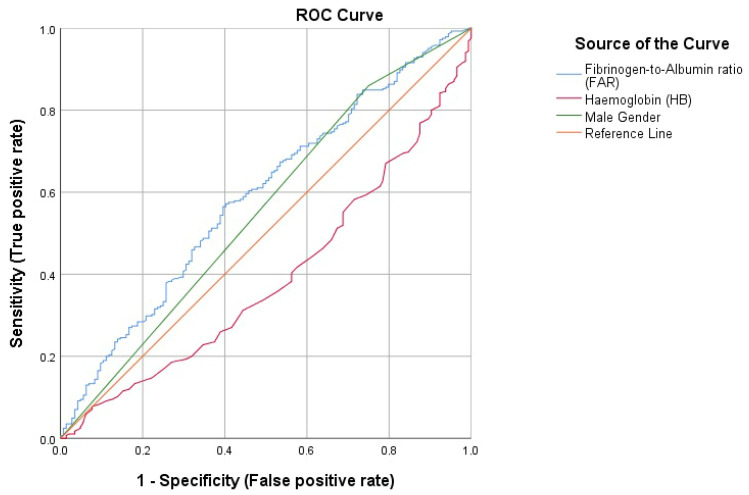
ROC curve for fibrinogen-to-albumin ratio (FAR), hemoglobin level (HB), and male gender. Diagonal segments are produced by ties. Reference line is the 45° diagonal line, serving as reference for a statistical test with no discriminatory ability.

ROC curve analysis reported an AUC of 0.595 (95% CI = 0.539–0.650; *p* = 0.001) for FAR which was significantly higher than for sex (AUC = 0.555, 95% CI = 0.496–0.614; *p* = 0.064) and hemoglobin level (AUC = 0.391, 95% CI = 0.336–0.446; *p* = 0.002) (Figure 1). According to the maximum Youden index value, the cut-off for FAR was set at 76, with a sensitivity of 0.573 and a specificity of 0.605. 

The univariate and multivariate logistic regression analyses are reported in Table 3. Increased age (OR = 1.040, 95% CI = 1.022–1.058; *p* < 0.0001) was associated, in the univariate analysis, with an increased risk of being diagnosed with any-grade BC, although the impact of this variable was very limited. Increased hemoglobin values, as well as increased albumin levels, were instead associated with decreased risk of an any-grade BC diagnosis, with OR = 0.826 (95% CI = 0.740–0.923; *p* = 0.001) and OR = 0.418 (95% CI = 0.288–0.803; *p* = 0.005), respectively. FAR was, although statistically significant, irrelevant when considered as a continuous variable (OR = 1.013, 95% CI = 1.004–1.022; *p* = 0.004), while when considered as a categorical variable, utilizing the previously reported cut-off, its impact was indeed notable (OR = 2.062, 95% CI = 1.378–3.084; *p* < 0.0001). Finally, males reported an increased risk of being diagnosed with BC 1.64-fold that of females (OR = 1.640, 95% CI = 1.045–2.572; *p* = 0.031). Despite the interesting results of the univariate analysis, in the multivariate analysis, only age (OR = 1.034, 95% CI = 1.014–1.055; *p* = 0.001), hemoglobin (OR = 0.814, 95% CI = 0.701–0.944; *p* = 0.006), and male gender (OR = 2.151, 95% CI = 1.253–3.694; *p* = 0.005) were statistically significant.

## 4. Discussion

Bladder cancer is the most common malignancy of the urinary tract, with urothelial carcinoma representing the most common histologic type [9]. Standardized treatment comprises transurethral resection (plus adjuvant intravesical chemotherapy or immunotherapy) for non-muscle-invasive tumors, while radical cystectomy is the gold standard approach for bladder-confined muscle-invasive tumors [23]. Although differences in grading do not modify the proposed treatment, the distinction between low grade and high grade has implications for the risk stratification and management of patients [24]. 

In an effort to improve the preoperative diagnosis of BC (without utilizing costly and limited available biomarkers), and considering the role of inflammation as a favorable factor in carcinogenesis, tumor progression, and metastasis, several systemic inflammatory markers have been investigated in relation to bladder cancer, comprising the neutrophil-to-lymphocyte ratio (NLR), platelet-to-lymphocyte ratio (PLR), lymphocyte-to-monocyte ratio (LMR), and neutrophil percentage-to-albumin ratio (NPAR) [25,26,27,28]. 

Recently, several pieces of evidence have outlined the role of fibrinogen in tumorigenesis due to its pathways involved in the epithelial–mesenchymal transition, cell proliferation, and angiogenesis, in addition to its consolidated role in the acute-phase response and inflammatory activities [18,29]. Albumin, conversely, represents not only a nutritional indicator, but is similarly involved in the anti-inflammatory response and is utilized as a prognostic predictor of cancer survival, in addition to being considered as a potential cancer drug carrier [30,31,32]. 

Due to these premises, the roles of albumin and fibrinogen as potential biomarkers in cancer have been investigated in different studies. Hwang et al. evaluated the prognostic influence of preoperative FAR in breast cancer, reporting a worse prognosis for patients with a higher FAR (>7.1) [21]. Analogously, a study by Xu et al. reported unfavorable overall survival in gall bladder cancer patients with FAR > 0.08 compared to patients with lower values [33]. Finally, An et al. reported high FAR (>7.75) as a worse prognostic factor in cervical cancer [20]. To date, one of the first studies to evaluate the role of preoperative FAR in bladder cancer patients was performed by Chen et al., who reported, similarly to other malignancies, worse overall survival and progression-free survival in MIBC patients with higher FAR (>0.08); in addition, they found better performance of FAR compared to NLR and PLR [34]. Similar results were also obtained, in another study by Claps et al., for the counterpart albumin-to-fibrinogen ratio (AFR), reporting, in patients with lower AFR, worse overall survival and cancer-specific survival [35,36]. In our study we sought to evaluate the role of FAR as a potential preoperative biomarker, investigating, in addition, its relation to histopathological results. As previously reported, we found an interesting correlation between FAR and different histopathological results as well as increased FAR and risk of BC diagnosis. This finding, in particular, was further confirmed when a proper cut-off was utilized. Although the relation between FAR and BC diagnosis was not further confirmed in the multivariate analysis, this could be related to the intrinsic nature of this novel biomarker, which is based on blood sample values only. The absence of other potential variables to be associated with FAR in our study could have, therefore, limited the consistency of this biomarker alone. Although this was not the aim of our study, we found an overall increased age in patients with LG and HG BC, in addition to an overall preponderance of male gender, confirmed in the logistic regression analysis as well. These data are consistent with the literature, which reports age and male gender as independent risk factors for BC, in addition to worse grade and the stage at diagnosis of elderly patients [5,37]. Regarding the decreased level of hemoglobin in LG and HG BC patients, compared to negative results, this finding could be related to the hematuria associated with malignancy. As a result, in the logistic regression analysis, increased hemoglobin serum values were associated with a decreased risk of BC diagnosis. Although we found no specific data regarding hemoglobin serum values in LG and HG BC, preoperative anemia in NMIBC is associated with worse cancer-specific and overall survival, in addition to increased rates of recurrence and progression [38]. Finally, differences in albumin and ALT serum values among negative, LG, and HG BC patients, although statistically significant, did not seem, in this case, to be clinically significant and should be further examined in larger studies. 

To the best of our knowledge, this study is one of the first to evaluate the role of preoperative FAR in the prediction of BC, particularly as a potential predictor of malignancy, as well as advanced-grade BC. We are, however, conscious of the different limitations of our work, and we comprehend, firstly, the retrospective nature of the study; secondly, the relatively small sample size of patients involved; and thirdly, the lack of follow-up, which impeded the evaluation of overall survival (OS), cancer-specific survival (CSS), progression-free survival (PFS), as well as recurrence-free survival (RFS), thus excluding the study of FAR as a prognostic factor. Another limitation is related to the absence of data regarding smoking status, which were incomplete due to the retrospective nature of the study. Finally, the absence of data regarding the multifocality of the tumors at the time of cystoscopy and TURBT represent another significant limitation. Although FAR could potentially reach a sensitivity superior to urine cytology (57% vs. 48%), further studies are required in order to provide a solid background for the use of this biomarker alone [39,40]. We instead recommend using FAR as an adjunctive biomarker aimed at aiding the discrimination of dubious diagnoses.

## 5. Conclusions

Preoperative high FAR could be an independent predictor of malignancy as well as advanced grade in patients with bladder cancer. Nevertheless, considering the limitations related to the absence of statistical significance in the multivariate analysis, as well as the limited sensitivity and specificity of this biomarker in the diagnosis of BC, this study has to be considered as an initial and pilot experience regarding the role of FAR as a diagnostic biomarker in BC. Further studies are required to confirm the potential role of FAR, alone and in combination with other diagnostic tools (i.e., urinary cytology), in the preoperative evaluation of potential bladder cancer.

## Figures and Tables

**Table 1:** Descriptive characteristics of patients involved (IQR: interquartile range).

	Median	IQR
**Age (years)**	73	14
**Hemoglobin (g/dL)**	14	2.2
**Creatinine (mg/dL)**	0.9	0.3
**Albumin (g/dL)**	4.3	0.5
**Uric acid (mg/dL)**	5.5	1.7
**Total Cholesterol (mg/dL)**	175	57
**Triglycerides (mg/dL)**	106	57
**LDL (mg/dL)**	109	52
**HDL (mg/dL)**	45	17
**AST (mU/mL)**	19	7
**ALT (mU/mL)**	17	13
**Fibrinogen (mg/dL)**	325	109.4
	**Count**	**Percentage**
**Gender**		
Male	413	81
Female	97	19
**Diabetes**		
Yes	78	15.3
No	432	84.7
**Chronic Kidney Disease**		
Yes	58	11.4
No	452	88.7
**Cancer**		
Yes	340	66.7
No	170	33.3
**Grade**		
Negative	170	33.3
Low-grade	152	29.8
High-grade	188	36.9
**Muscle-Invasive Bladder Cancer**		
Yes	59	17.8
No	272	82.2

**Table 2:** Preoperative data according to histopathological analysis (IQR: interquartile range).

	Normal Range	Negative		Low-Grade		High-Grade		*p* Value
		Median	IQR	Median	IQR	Median	IQR	
**Age (years)**	N.A	70	15	72	15	75	13	**<0.0001**
**Hemoglobin (g/dL)**	12–17.5	14.5	2.13	14	2.3	13.6	2.32	**<0.0001**
**Creatinine (mg/dL)**	0.7–1.2	0.9	0.33	0.9	0.3	0.92	0.36	**0.008**
**Albumin (g/dL)**	3.2–4.6	4.4	0.5	4.4	0.4	4.3	0.5	**0.021**
**Uric acid (mg/dL)**	3.5–7.2	5.3	1.8	5.8	1.8	5.7	2	0.072
**Total Cholesterol (mg/dL)**	<200	178	48	176	63	167.5	60	0.344
**Triglycerides (mg/dL)**	<150	95.5	58	110	51	107	66	0.218
**LDL (mg/dL)**	<100	113	45	105	63	108	54	0.572
**HDL (mg/dL)**	>40	47	15	45	21	44	16	0.118
**AST (mU/mL)**	0–34	19	7	19	7	19	8	0.852
**ALT (mU/mL)**	0–55	19	12	17	12	16	10	**0.004**
**Fibrinogen (mg/dL)**	160–350	311	108.5	338	108	326	121.3	0.089
**Fibrinogen-to-Albumin Ratio (FAR)**	N.A	70.55	28.19	78.2	25.87	81.01	31.43	**0.006**
		Count	Percentage	Count	Percentage	Count	Percentage	
**Gender (male)**	N.A	127	74.7	124	81.6	162	86.2	**0.022**
**Diabetes (yes)**	N.A	20	13.1	19	13.9	39	23.2	**0.027**
**Chronic Kidney Disease (yes)**	N.A	15	19.5	15	19.7	28	30.1	0.170

**Table 3:** Univariate and multivariate logistic regression analysis.

Variable	Univariate		Multivariate	
	OR (95% CI)	*p* value	OR (95% CI)	*p* value
**Age (years)**	1.040 (1.022–1.058)	**<0.0001**	1.034 (1.014–1.055)	**0.001**
**Hemoglobin (g/dL)**	0.826 (0.740–0.923)	**0.001**	0.814 (0.701–0.944)	**0.006**
**Creatinine (mg/dL)**	0.957 (0.866–1.058)	0.392	0.906 (0.740–1.109)	0.906
**Albumin (g/dL)**	0.481 (0.288–0.803)	**0.005**	1.165 (0.594–2.285)	0.658
**ALT (mU/mL)**	0.997 (0.980–1.013)	0.674	1.011 (0.991–1.032)	0.264
**FAR continuous**	1.013 (1.004–1.022)	**0.004**	1.001 (0.988–1.013)	0.928
**FAR categorical**				
<76	Ref.	-	Ref.	-
>76	2.062 (1.378–3.084)	**<0.0001**	1.657 (0.895–3.066)	0.108
**Gender**				
Female	Ref.	-	Ref.	-
Male	1.640 (1.045–2.572)	**0.031**	2.151 (1.253–3.694)	**0.005**
**Diabetes**				
No	Ref.	-	Ref.	-
Yes	1.171 (0.988–2.963)	0.055	1.471 (0.793–2.728)	0.221

## Data Availability

Data available on request.
